# Physical activity patterns, genetic susceptibility, and incident ischemic stroke: a population-based cohort study

**DOI:** 10.3389/fnagi.2026.1840853

**Published:** 2026-05-29

**Authors:** Zheng Tu, Caixiang Zhuang, Yuwen Hu, Jianghai He, Ziyang Jin, Haoxiang Hu, Yunhan Zhao, Yanyan Zheng

**Affiliations:** 1 Department of Neurology, Postgraduate Training Base Alliance of Wenzhou Medical University (Wenzhou People’s Hospital), Wenzhou, China; 2 Department of Neurology, First Affiliated Hospital of Wenzhou Medical University, Wenzhou, China; 3 Wenzhou Third Clinical Institute Affiliated to Wenzhou Medical University, Wenzhou People’s Hospital, Wenzhou, China

**Keywords:** ischemic stroke, physical activity pattern, polygenic risk score (PRS), UK Biobank, weekend warrior

## Abstract

**Background:**

Ischemic stroke is a leading cause of death and disability worldwide. Whether different weekly physical activity patterns confer similar protection remains unclear, and genetic susceptibility may further influence risk. We therefore examined the associations of physical activity pattern, polygenic risk, and their joint categories with incident ischemic stroke.

**Methods:**

In this prospective UK Biobank analysis, 84,006 participants with valid accelerometer data were classified by weekly moderate-to-vigorous physical activity (MVPA) pattern (inactive, active regular, or weekend warrior) and polygenic risk score (PRS) category (low, intermediate, or high). Cox models were used to estimate HRs and 95% CIs for incident ischemic stroke, including joint analyses across nine combined categories.

**Results:**

At ≥150 min/week of MVPA, both weekend warrior and active regular patterns were associated with lower ischemic stroke risk than inactivity, with no statistically significant difference observed between the two active patterns. High, but not intermediate, polygenic risk was associated with increased risk (HR 1.83, 95% CI 1.35–2.47). In joint analyses, excess risk was evident only in inactive participants with intermediate or high genetic risk.

**Conclusion:**

Physical activity pattern and PRS were both associated with incident ischemic stroke. Meeting weekly MVPA recommendations, whether regularly or as a weekend warrior, was associated with lower risk, whereas higher genetic risk was associated with greater risk.

## Introduction

Worldwide, stroke continues to impose a major burden in terms of death and long-term disability (GBD 2021 Stroke Risk Factor Collaborators, 2024). Recent global estimates suggest that ischemic stroke represents the majority of stroke cases and contributes markedly to the worldwide burden of neurological disability. Ischemic stroke is not only a major vascular disorder but also an important aging-related neurovascular condition that substantially contributes to cognitive decline, neurological disability, and loss of functional independence in older adults. As population aging accelerates worldwide, identifying modifiable behavioral factors associated with brain and cerebrovascular health has become increasingly important in aging neuroscience research (Feigin et al., 2025).

Physical activity is a well-established modifiable factor in stroke prevention (De Santis et al., 2024). Current guidelines recommend that adults accumulate at least 150–300 min of moderate-intensity physical activity, 75–150 min of vigorous-intensity physical activity, or an equivalent combination each week, collectively referred to as moderate-to-vigorous physical activity (MVPA) (Bull et al., 2020). Prospective studies have demonstrated that higher levels of physical activity are associated with a reduced risk of stroke (Khurshid et al., 2023). However, whether the temporal distribution of weekly physical activity provides prognostic information beyond total activity volume remains uncertain (Yang et al., 2025).

This is particularly pertinent to people who perform the majority of their weekly physical activity over only one or 2 days, often termed the “weekend warrior” pattern. Recent accelerometer-based research indicates that when the overall weekly activity volume is adequate, such patterns may provide cardiovascular benefits comparable to those achieved with more evenly distributed activity (Khurshid et al., 2023). In addition to lifestyle factors, genetic susceptibility also contributes to the risk of ischemic stroke (Huang et al., 2024; Kim et al., 2024). Polygenic risk scores (PRSs) provide an approach for quantifying cumulative genetic susceptibility to complex diseases, including stroke (Marini et al., 2024; Yang et al., 2024). Emerging evidence also suggests that favorable health-related behaviors may be associated with lower vascular risk even among individuals with higher genetic susceptibility (Lu et al., 2021).

However, whether the weekend warrior pattern provides comparable protection against incident ischemic stroke remains insufficiently understood. Unlike broad cardiovascular outcomes, ischemic stroke is a major neurovascular endpoint associated with substantial long-term disability and public health burden (Feigin et al., 2025). Its occurrence may be influenced by both modifiable lifestyle behaviours and inherited susceptibility, making it important to evaluate physical activity patterns together with genetic risk (Mishra et al., 2022). Clarifying whether concentrated MVPA is associated with lower ischemic stroke risk may provide pragmatic evidence for individuals who are unable to distribute activity evenly throughout the week. Such evidence may also help refine lifestyle-based prevention strategies for populations with different levels of genetic susceptibility.

Nevertheless, few studies have jointly evaluated objectively measured physical activity patterns and polygenic susceptibility in relation to incident ischemic stroke. Based on accelerometer measurements from the UK Biobank, we investigated the separate and combined relationships of physical activity patterns and polygenic risk with incident ischemic stroke. We expected that participants with either regular or weekend warrior activity patterns would have a lower risk of stroke than inactive individuals, that elevated polygenic risk would be associated with greater stroke risk, and that adequate physical activity would be associated with lower ischemic stroke risk even among individuals with higher genetic susceptibility.

## Methods

### Study design and participants

Data for this prospective cohort analysis were drawn from the UK Biobank, a large population-based cohort comprising over 500,000 adults aged 40–69 years recruited throughout the United Kingdom between 2006 and 2010. At baseline, participants provided information through questionnaires and interviews, along with physical assessments, and their data were linked to national health records. All participants provided written informed consent, and ethical approval for the study was granted by the relevant research ethics committee.

**Figure 1:**
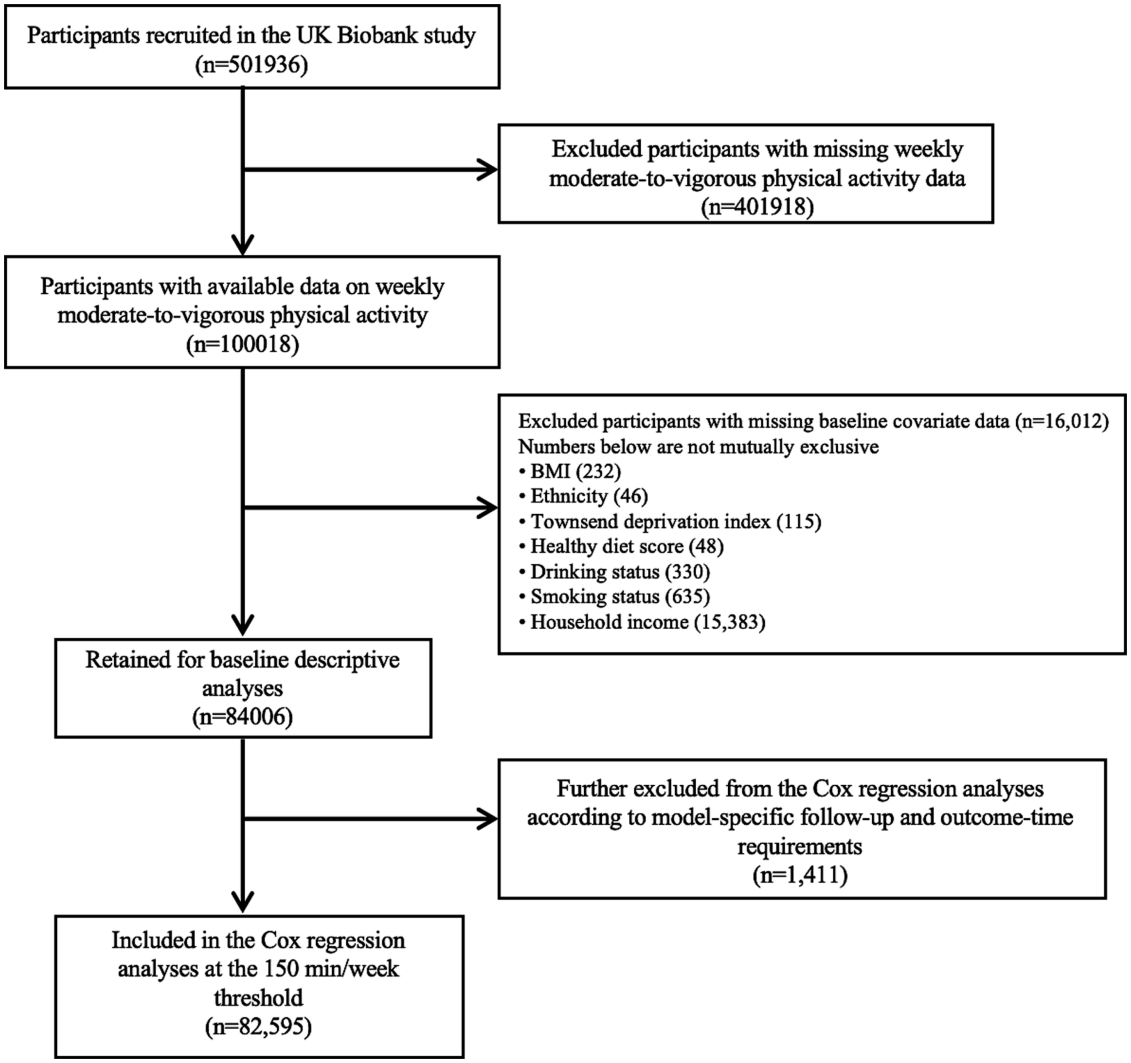
Flowchart of participant selection for the present study. The diagram summarizes the inclusion and exclusion process of UK Biobank participants in the final analysis.

For this analysis, we selected participants with available accelerometer-derived physical activity measurements. Participants were excluded if valid weekly moderate-to-vigorous physical activity (MVPA) data were unavailable or if baseline covariate information was incomplete. After these exclusions, 84,006 participants remained for the final analysis. A complete-case analysis was performed; participants with missing weekly MVPA data or missing baseline covariate information were excluded from the final analytic cohort. Follow-up started from the date of accelerometer assessment and continued until the first occurrence of ischemic stroke, death, loss to follow-up, or the end of linked health-record follow-up available in the UK Biobank, whichever came first. The flow of participant selection, including exclusions related to missing MVPA data and incomplete baseline covariates, is shown in Figure 1.

### Assessment of physical activity pattern

Accelerometer-derived measurements from the wrist-worn triaxial Axivity AX3 device were used to evaluate physical activity in the UK Biobank. In the accelerometer sub-study, participants wore the device continuously for 7 days, and the raw acceleration signals were processed to derive physical activity measures. Previous reports from the UK Biobank accelerometer project have described the device characteristics and data-processing procedures in detail.

In this study, weekly MVPA and its day-to-day distribution were derived from accelerometer data. In accordance with the World Health Organization recommendation, 150 min/week of MVPA was used as the primary threshold for defining sufficient physical activity.

Participants were classified into three groups based on total weekly MVPA and how it was distributed across the week:

1. Inactive, with total weekly MVPA <150 min/week.
2. Active regular, achieving ≥150 min/week of MVPA with <50% of total activity accumulated during the two most active days.
3. Active weekend warrior, achieving ≥150 min/week of MVPA with ≥50% of total activity concentrated in the two most active days.

In sensitivity analyses, alternative thresholds for weekly MVPA were also examined, including 101, 230, and 403 min/week. These cutoffs were selected with reference to previous UK Biobank accelerometer-based research in which these values were used as distribution-based MVPA thresholds to assess the stability of findings across different activity definitions (Lin et al., 2024). These thresholds were not newly derived from the distribution of the final analytic sample in the present study, but were applied as literature-based alternative definitions to evaluate the robustness of the observed associations.

### Ascertainment of incident ischemic stroke and follow-up

The outcome of interest was incident ischemic stroke. Incident ischemic stroke was ascertained from the UK Biobank first-occurrence records using the ICD-10 code I63 for ischemic stroke. Only ischemic stroke events occurring after the baseline accelerometer assessment were considered incident outcomes; stroke records before or at baseline were not counted as incident ischemic stroke events. Participants without incident ischemic stroke were censored at death, loss to follow-up, or the end of linked health-record follow-up available in the UK Biobank. No additional exclusion was applied for other major cardiovascular diseases at baseline.

### Polygenic risk score

Genetic susceptibility to ischemic stroke was assessed using the enhanced ischemic stroke polygenic risk score (PRS) provided by the UK Biobank. Specifically, we used the UK Biobank-released enhanced PRS for ischaemic stroke (ISS), corresponding to UK Biobank Data-Field 26,249. This precomputed PRS was used as a genome-wide summary measure of inherited susceptibility to ischemic stroke, and no *de novo* PRS construction was performed in the present study.

The PRS was examined both as a continuous measure and in categorical form. In the categorical analysis, PRS values were divided into tertiles to classify participants as having low, intermediate, or high genetic risk. This categorization approach was used to facilitate interpretation and maintain adequate numbers of participants and outcome events within each category, and has been commonly adopted in previous PRS-based epidemiological studies (Neumann et al., 2021; Small et al., 2024). For joint analyses, the three PRS categories were combined with the three physical activity pattern categories to generate nine mutually exclusive groups. Accordingly, PRS-related analyses were restricted to participants with available polygenic risk score data, and participants without PRS information were not included in these analyses.

Because PRS analyses may be affected by population structure and ancestry differences, the interpretation of the PRS findings was restricted to risk stratification rather than clinical prediction. Self-reported ethnicity was included as a covariate in multivariable models; however, genetic principal components, genotyping array, and relatedness were not additionally incorporated into the Cox models in the present analysis.

### Statistical analysis

Baseline demographic and clinical features were described separately for each physical activity pattern group. Continuous data are reported in terms of median and interquartile range, while categorical data are expressed as frequency distributions and proportions. Comparisons across groups were carried out with nonparametric methods for continuous variables and chi-square procedures for categorical variables, as appropriate.

The associations of physical activity pattern, PRS, and their joint groupings with incident ischemic stroke were assessed with Cox regression models, and the results are presented as HRs with corresponding 95% CIs. In analyses focused on physical activity pattern, the first model included only age and sex, while the fully adjusted model additionally incorporated BMI, ethnicity, Townsend deprivation index (TDI), healthy diet score, educational attainment, drinking status, smoking status, and household income. For pairwise comparisons among activity categories, the inactive group and the active regular group were alternately treated as the reference group in separate models.

In the PRS analyses, age and sex were included in Model 1. Model 2 additionally adjusted for BMI, ethnicity, TDI, healthy diet score, educational attainment, drinking status, smoking status, and household income, and Model 3 further included physical activity pattern. PRS was examined in both continuous form and categorical form according to tertile distribution. For joint analyses, nine categories generated from PRS tertiles and physical activity pattern were entered into Cox models, with the low genetic risk and active regular group treated as the reference category. These joint-category analyses were used to describe the combined associations of physical activity pattern and polygenic risk with incident ischemic stroke. No formal interaction term between physical activity pattern and PRS was included in the Cox models; therefore, a *p* value for interaction was not estimated. Because PRS-related analyses required available genetic data, these analyses were restricted to participants with available polygenic risk score data. Analytic samples were therefore model-specific. The PRS-only analysis included participants with available PRS data and the required covariate, follow-up, and outcome-time information. The joint PRS–physical activity analysis additionally required available physical activity pattern data. Therefore, the numbers of participants and ischemic stroke events in the PRS-related analyses were smaller than those in the main physical activity analysis.

To illustrate differences in activity accumulation patterns, density plots were constructed to compare the distribution of MVPA performed during the two most active days with that accumulated during the remaining 5 days in the active regular and active weekend warrior groups. All analyses were carried out in R, and statistical significance was inferred at a two-tailed threshold of *p* < 0.05.

## Results

### Study population and baseline characteristics

After excluding participants with missing weekly MVPA data and baseline covariate data, 84,006 participants were retained for baseline descriptive analyses, among whom 1,179 incident ischemic stroke events were identified during follow-up. According to weekly MVPA, these participants were grouped as inactive (*n* = 31,653), active regular (*n* = 17,410), or active weekend warrior (WW) (*n* = 34,943). For the multivariable Cox regression analyses at the guideline-based 150 min/week threshold, participants were further required to meet follow-up and outcome-time requirements. Accordingly, the Cox regression analytic sample included 82,595 participants and 1,147 incident ischemic stroke events, comprising 30,970 inactive participants with 510 events, 17,176 active regular participants with 213 events, and 34,449 active WW participants with 424 events.

Table 1 summarizes baseline characteristics across physical activity pattern groups among the 84,006 participants retained for baseline descriptive analyses. Compared with inactive participants, those classified as active weekend warrior (WW) or active regular were generally younger, less often women, and less likely to be obese. The three groups also differed significantly with respect to multiple other socioeconomic and lifestyle characteristics. Of note, never drinking was more common in the active WW group than in the inactive and active regular groups. Weekly MVPA was highest in the active regular group.

**Table 1:** Baseline characteristics of overall participants by physical activity pattern.

Variables	Characteristics of overall participants			
	Active regular	Inactive	Active WW	*p*-value
No.	17,410	31,653	34,943	
Age median (Q1, Q3)	56 (49, 61)	58 (51, 63)	57 (50, 62)	<0.001
Gender (%)				<0.001
Female	8,841 (50.78)	20,590 (65.05)	17,725 (50.73)	
Male	8,569 (49.22)	11,063 (34.95)	17,218 (49.27)	
BMI (%)				<0.001
Underweight	160 (0.92)	115 (0.36)	191 (0.55)	
Normal	8,096 (46.50)	9,717 (30.70)	14,718 (42.12)	
Overweight	6,861 (39.41)	12,795 (40.42)	14,858 (42.52)	
Obese	2,293 (13.17)	9,026 (28.52)	5,176 (14.81)	
Ethnicity (%)				<0.001
Non white	615 (3.53)	1,277 (4.03)	990 (2.83)	
White	16,795 (96.47)	30,376 (95.97)	33,953 (97.17)	<0.001
TDI median (Q1, Q3)	−2.06 (−3.62, 0.52)	−2.46 (−3.80, −0.21)	−2.58 (−3.90, −0.48)	<0.001
Score diet Median (Q1, Q3)	3.00 (2.00, 4.00)	3.00 (2.00, 4.00)	3.00 (2.00, 4.00)	
Education (%)				0.766
UnKnown	2,710 (15.57)	4,819 (15.22)	5,426 (15.53)	
College	6,087 (34.96)	11,156 (35.24)	12,174 (34.84)	
Other	8,613 (49.47)	15,678 (49.53)	17,343 (49.63)	
Drinking status (%)				0.304
Never	610 (3.50)	1,198 (3.78)	1,396 (4.00)	
Previous	610 (3.50)	1,086 (3.43)	1,258 (3.60)	
Current	16,190 (92.99)	29,369 (92.78)	32,289 (92.40)	
Smoking status (%)				0.800
Never	9,455 (54.31)	17,256 (54.52)	19,081 (54.61)	
Previous	6,104 (35.06)	11,083 (35.01)	12,181 (34.86)	
Current	1,851 (10.63)	3,314 (10.47)	3,681 (10.53)	
Household income (%)				0.175
Less than 18,000	3,999 (22.97)	7,075 (22.35)	8,067 (23.09)	
18,000 to 30,999	4,454 (25.58)	8,078 (25.52)	8,738 (25.01)	
31,000 to 51,999	4,578 (26.30)	8,258 (26.09)	9,184 (26.28)	
52,000 to 100,000	3,470 (19.93)	6,499 (20.53)	7,041 (20.15)	
Greater than 100,000	909 (5.22)	1,743 (5.51)	1,913 (5.47)	

Table 1 presents baseline characteristics of the 84,006 participants retained for baseline descriptive analyses after exclusion of missing weekly MVPA and baseline covariate data. Abbreviations: BMI, body mass index; TDI, Townsend deprivation index; MVPA, moderate-to-vigorous physical activity. The MVPA threshold was 150 min per week. Data are presented as median (interquartile range) or number (percentage), as appropriate. Continuous variables were compared using the Kruskal–Wallis test, and categorical variables were compared using the chi-square test.

Overall, participants with sufficient weekly MVPA appeared to have a more favorable socioeconomic and lifestyle profile than inactive individuals.

### Distribution of weekly MVPA across physical activity patterns

**Figure 2:**
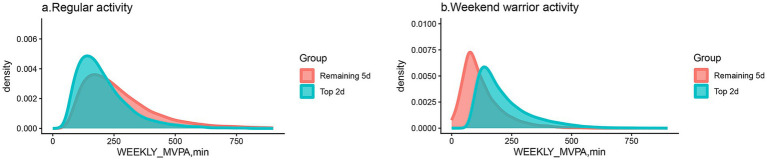
Distribution of weekly MVPA across the two most active days and the remaining 5 days. Weekly distribution of moderate-to-vigorous physical activity (MVPA) between the two most active days and the remaining 5 days among individuals achieving ≥150 min/week of MVPA. Regular activity **(a)** and weekend warrior activity **(b)** patterns are shown. The top 2 days represent the summed MVPA minutes from the two most active days in a week, whereas the remaining 5 days represent the accumulated MVPA minutes during the other days. The *y*-axis shows probability density, indicating the relative distribution of participants across MVPA levels.

The density plots of weekly MVPA distribution are presented in Figure 2. Among participants classified as active regular, MVPA was distributed more evenly across the week, whereas in the active WW group, a greater proportion of weekly MVPA was accumulated during the two most active days. The observed distribution supported the predefined grouping of participants into the active regular and active WW categories.

### Associations between physical activity patterns and incident ischemic stroke

**Figure 3:**
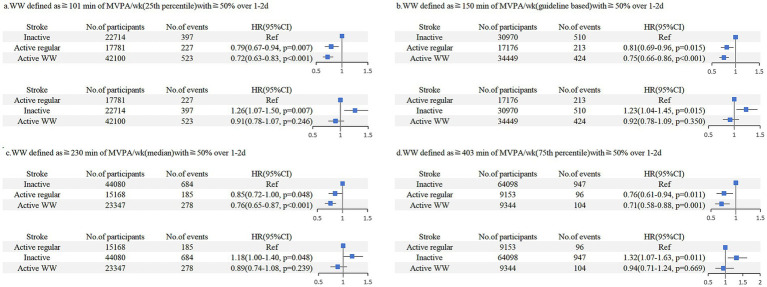
Associations of physical activity patterns with incident ischemic stroke. Hazard ratios (HRs) and 95% confidence intervals (CIs) for incident ischemic stroke are shown for the inactive, active regular, and active weekend warrior groups. Panels **a–d** show the results using weekend warrior definitions based on ≥101, ≥150, ≥230, and ≥403 min of MVPA/week, respectively, with ≥50% of weekly MVPA accumulated over 1–2 days. Separate multivariable Cox regression analyses were performed using either the inactive group or the active regular group as the reference category. The numbers shown in the embedded tables correspond to the participants included in each Cox regression analysis after applying follow-up and outcome-time requirements.

Figure 3 shows the risk estimates for incident ischemic stroke across physical activity pattern groups.

Relative to the inactive group, participants in the active regular and active WW categories, both meeting the criterion of ≥150 min/week of MVPA, had reduced risks of incident ischemic stroke in the age- and sex-adjusted analysis. In the multivariable-adjusted model, the HR was 0.81 (95% CI, 0.69–0.96) for the active regular group and 0.75 (95% CI, 0.66–0.86) for the active WW group.

When the active regular group served as the reference, participants in the inactive group exhibited a higher risk of incident ischemic stroke (HR = 1.23, 95% CI 1.04–1.45). By comparison, no statistically significant difference was found between the active weekend warrior (WW) and active regular groups (HR = 0.92, 95% CI 0.78–1.09; *p* = 0.350).

These findings suggest that both active weekend warrior and active regular patterns were associated with lower ischemic stroke risk compared with inactivity, while no statistically significant difference was observed between the two active groups.

### Risk of incident ischemic stroke according to polygenic risk score category

The associations between polygenic risk categories and incident ischemic stroke are shown in Table 2. This PRS-only analysis was restricted to participants with available polygenic risk score data and required model information, comprising 21,011 participants and 262 incident ischemic stroke events. Compared with the low genetic risk group, the intermediate-risk group did not show a statistically significant elevation in incident ischemic stroke risk, whereas the high-risk group exhibited a significantly higher risk. After full adjustment, the HRs were 1.25 (95% CI, 0.91–1.72) and 1.83 (95% CI, 1.35–2.47), respectively.

**Table 2:** Association between polygenic risk score (PRS) and incident ischemic stroke in the UK biobank.

Polygenic risk score	Events	Total/person years	Model 1		Model 2		Model 3	
			HR (95% CI)	*p*-value	HR (95% CI)	*p*-value	HR (95% CI)	*p*-value
Low genetic risk	71	7581/60070	Ref		Ref		Ref	
Intermediate genetic risk	82	7000/55694	1.24 (0.90, 1.70)	0.189	1.25 (0.91, 1.72)	0.168	1.25 (0.91, 1.72)	0.173
High genetic risk	109	6430/51227	1.84 (1.36, 2.48)	6.58 × 10^−5^	1.84 (1.36, 2.49)	6.93 × 10^−5^	1.83 (1.35, 2.47)	8.21 × 10^−5^

**Figure 4:**
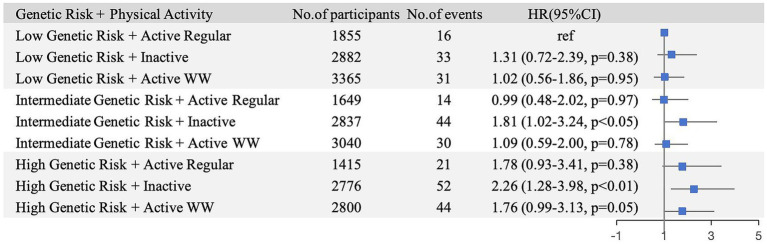
Joint associations of physical activity patterns and polygenic risk with incident ischemic stroke. Hazard ratios (HRs) and 95% confidence intervals (CIs) for incident ischemic stroke are shown across joint categories of physical activity pattern and polygenic risk at the 150 min/week threshold. The low genetic risk + active regular group was used as the reference category. The joint analysis was restricted to participants with available data on both polygenic risk score and physical activity pattern. The embedded table represents the joint PRS–physical activity analytic sample, including 22,619 participants and 285 incident ischemic stroke events. No formal interaction term between physical activity pattern and polygenic risk score was included in the Cox models; therefore, these estimates should be interpreted as descriptive joint associations rather than evidence of statistically significant effect modification.

This table presents the PRS-only Cox regression analysis, which was restricted to participants with available polygenic risk score data and required covariate, follow-up, and outcome-time information. The analysis included 21,011 participants and 262 incident ischemic stroke events. This analysis is distinct from the joint PRS–physical activity analysis shown in Figure 4. Model 1 was adjusted for age and sex. Model 2 was further adjusted for body mass index, ethnicity, Townsend deprivation index, diet score, education level, smoking status, drinking status, and household income. Model 3 was additionally adjusted for physical activity pattern. HRs and 95% CIs were estimated using Cox proportional hazards models. PRS was analysed as both a continuous variable and a categorical variable (low, intermediate, and high genetic risk), with the low genetic risk group as the reference.

### Combined associations of physical activity patterns and polygenic susceptibility with ischemic stroke

Figure 4 shows the associations of combined physical activity pattern and polygenic risk categories with incident ischemic stroke at the 150 min/week threshold. This joint PRS–physical activity analysis was restricted to participants with available data on both polygenic risk score and physical activity pattern, and included 22,619 participants and 285 incident ischemic stroke events. With the [Low Genetic Risk + Active Regular] group as the reference category, significantly increased risks of incident ischemic stroke were observed in the [Intermediate Genetic Risk + Inactive] and [High Genetic Risk + Inactive] groups, with the highest risk observed in the [High Genetic Risk + Inactive] group (HR = 2.26, 95% CI = 1.28–3.98). The other combined categories showed elevated or directionally similar estimates, but most associations were not statistically significant. In particular, among participants with high genetic risk, the active regular and active WW categories had HRs above 1.0 compared with the reference group, and their confidence intervals crossed the null value. Therefore, these results do not demonstrate a statistically significant protective association of physical activity patterns within the high genetic risk stratum. No formal p-interaction between physical activity pattern and PRS was estimated; therefore, these joint-category findings should be interpreted as descriptive combined associations rather than evidence of statistically significant effect modification.

### Additional analyses using alternative MVPA thresholds

To assess the robustness of the findings, joint analyses were repeated using alternative MVPA thresholds of 101, 230, and 403 min/week, with the corresponding results shown in Supplementary Tables S1–S3. Although the specific joint categories reaching statistical significance differed across thresholds, the overall pattern was similar. In general, combinations characterized by lower physical activity and higher genetic risk tended to show less favorable associations with incident ischemic stroke. These findings indicate that the combined association of physical activity pattern and polygenic risk was generally consistent across different MVPA thresholds.

## Discussion

This prospective cohort analysis of UK Biobank participants with accelerometer-measured physical activity data examined the separate and joint associations of physical activity pattern and polygenic susceptibility with incident ischemic stroke. Relative to inactive individuals, participants in the active regular and active weekend warrior groups showed a lower risk of ischemic stroke. No statistically significant difference was observed between the two active patterns. A significantly elevated risk was observed in the high genetic risk group, whereas no significant association was identified for the intermediate-risk group. In the joint-category analyses, physically inactive participants with intermediate or high genetic risk tended to have the highest risk estimates. However, the active regular and active weekend warrior categories within the high genetic risk stratum did not show statistically significant protective associations, as their confidence intervals crossed the null value. Therefore, these joint-category findings should be interpreted cautiously as descriptive combined associations rather than evidence that physical activity clearly offsets high genetic susceptibility.

Our findings regarding physical activity patterns are broadly consistent with recent accelerometer-based studies. These studies suggest that, among individuals achieving guideline-recommended weekly moderate-to-vigorous physical activity (MVPA), concentrating activity within 1–2 days is associated with lower risks of adverse health outcomes, with no clear evidence of major differences compared with more evenly distributed activity patterns. A phenome-wide analysis in the UK Biobank cohort, comprising 89,573 participants, showed that both weekend warrior and regular activity patterns were associated with a lower risk of more than 200 incident diseases, with particularly strong associations observed for cardiometabolic conditions. No significant differences were found in direct comparisons between the two patterns (Kany et al., 2024). Similarly, evidence from a dual-cohort study including the UK Biobank and NHANES indicated that, once weekly MVPA reached ≥150 min, both weekend warrior and regular activity patterns were associated with lower risks of all-cause, cardiovascular, and cancer mortality compared with inactive behavior, without statistically significant differences between the two active patterns. These findings provide context for our observation that both active regular and active weekend warrior patterns were associated with lower ischemic stroke risk than inactivity, whereas the two active patterns did not differ significantly from each other. However, previous studies mainly focused on activity patterns or total MVPA volume, whereas the present study further examined accelerometer-derived activity patterns in combination with genetic susceptibility (Ren et al., 2025).

The association between higher polygenic risk and incident ischemic stroke observed in our study is consistent with accumulating evidence that genome-wide polygenic scores can capture part of the inherited susceptibility to stroke (Neumann et al., 2021). In a large primary prevention analysis from the Million Veteran Program, ischemic stroke polygenic risk scores were also associated with incident ischemic stroke, although their incremental predictive value beyond traditional clinical predictors was modest. More recently, data from the China Kadoorie Biobank and UK Biobank further supported the relevance of polygenic risk in stroke susceptibility, showing that integrated polygenic scores were associated not only with first incident stroke but also with subsequent vascular outcomes among stroke survivors (Vassy et al., 2023; Han et al., 2025). These findings provide a rationale for considering genetic susceptibility together with modifiable lifestyle patterns when evaluating ischemic stroke risk.

Previous studies have demonstrated the feasibility and value of accelerometer-derived physical activity assessment in large population-based cohorts and its application to prospective analyses of cardiovascular outcomes (Doherty et al., 2017; Li et al., 2025). Building on these separate associations, the present study further examined accelerometer-derived physical activity patterns together with polygenic susceptibility in relation to incident ischemic stroke. Rather than demonstrating improved prediction, our joint-category analysis provides descriptive evidence on how objectively measured activity patterns and inherited susceptibility coexist in relation to ischemic stroke risk. Accelerometer-based studies have mainly focused on whether weekend warrior and regular activity patterns differ in relation to cardiovascular outcomes, whereas polygenic-score studies have mainly focused on the independent or incremental value of genetic risk prediction for stroke (O’Donnell et al., 2016; Abraham et al., 2019). Previous studies have highlighted the potential value of polygenic scores for risk stratification and prevention-oriented research, while also emphasizing that genetic susceptibility should be interpreted together with clinical, environmental, and lifestyle factors (Torkamani et al., 2018; Khera et al., 2019). In our analysis, physically inactive participants with intermediate or high genetic risk tended to have the highest risk estimates. However, the active regular and active weekend warrior categories within the high genetic risk tier were not statistically significant, and their confidence intervals crossed the null value. These results therefore should not be interpreted as demonstrating a clear protective association of physical activity among participants with high genetic susceptibility. Therefore, the present study adds to the literature by jointly evaluating objectively measured physical activity patterns and polygenic susceptibility in relation to incident ischemic stroke, while interpreting these results cautiously as joint associations rather than evidence of statistical interaction or attenuation of genetic risk.

From an aging-neuroscience perspective, ischemic stroke remains an important contributor to long-term neurological disability and functional decline in later life; therefore, clarifying how objectively measured physical activity patterns relate to ischemic stroke risk may have implications for preserving brain health during aging (Feigin et al., 2025). This is also relevant to public health guidance, as current recommendations emphasize achieving at least 150 min of moderate-to-vigorous physical activity per week without requiring that activity be distributed evenly across the week (Bull et al., 2020). In parallel, polygenic-risk score research has raised the possibility of identifying individuals with elevated inherited susceptibility before clinical disease develops, although the clinical utility of such approaches remains under evaluation. Against this background, our findings suggest that integrating accelerometer-derived physical activity patterns with polygenic susceptibility may help characterize joint risk profiles for ischemic stroke, particularly by identifying physically inactive individuals with higher genetic risk estimates. However, this should be viewed as a risk-assessment perspective rather than evidence that physical activity offsets genetic susceptibility. Beyond risk stratification, these findings may also be considered in the context of neurobiological mechanisms linking physical activity to cerebrovascular health.

Although the present study was not designed to directly examine neurobiological mechanisms, several plausible pathways may link sufficient physical activity with lower ischemic stroke risk. Regular physical activity has been associated with improved cerebrovascular function, endothelial health, and neurovascular regulation, which may contribute to healthier cerebrovascular aging (Tomoto and Zhang, 2024). Experimental and translational studies also suggest that exercise may reduce systemic and neurovascular inflammation, preserve blood–brain barrier integrity, and attenuate neurovascular unit injury after cerebral ischemia (Malkiewicz et al., 2019; Zhang et al., 2022). These mechanisms may be particularly relevant to ischemic stroke, in which vascular inflammation, blood–brain barrier dysfunction, and neuroinflammatory responses contribute to cerebrovascular injury and post-stroke neurological impairment (Candelario-Jalil et al., 2022). Therefore, our epidemiological findings may be viewed as complementary to mechanistic evidence linking physical activity to cerebrovascular and neurobiological resilience. However, because inflammatory markers, blood–brain barrier measures, and neuroimaging indicators of neurovascular injury were not assessed in the present study, these mechanistic interpretations remain hypothesis-generating and require further investigation.

The results of this study should be interpreted in light of several limitations. First, physical activity was assessed over a single one-week accelerometer monitoring period, which may not fully capture long-term habitual activity throughout follow-up (Troiano et al., 2014). Although one-week accelerometer measurements have been widely used in large population-based cohort studies, including the UK Biobank, changes in physical activity over time may have led to exposure misclassification (Doherty et al., 2017). Because physical activity behaviours may change over time, some degree of exposure misclassification is unavoidable. In addition, the classification of MVPA depends on operational thresholds and accelerometer processing criteria, and previous studies have shown that estimates may vary according to different accelerometer-based cutoffs and analytical approaches (Migueles et al., 2017). Such measurement error may have attenuated the observed associations toward the null. In addition, reverse causation cannot be completely excluded. Although participants with a history of stroke at baseline were excluded and physical activity was assessed before incident ischemic stroke events, preclinical cerebrovascular disease, frailty, or other underlying health conditions before diagnosis may have reduced physical activity levels. We did not perform a sensitivity analysis excluding ischemic stroke events occurring within the first 2 years after accelerometer assessment; therefore, the possibility that early events may have influenced the observed associations cannot be fully ruled out. Future studies with repeated physical activity assessments and landmark sensitivity analyses are needed to further evaluate this issue. Second, residual confounding cannot be excluded in observational analyses even after multivariable adjustment (Hernan and Robins, 2016). In the present study, the proportion of female participants was higher in the inactive group. Although sex was included as a covariate in all multivariable models, previous studies have suggested that physical activity behaviours and cardiovascular risk profiles may differ between men and women, and therefore residual confounding related to sex-specific activity patterns cannot be completely excluded (Rajendran et al., 2023). In addition, drinking status differed across physical activity pattern groups, with a higher proportion of never drinkers observed in the weekend warrior group. Although drinking status was adjusted for in the multivariable models, residual confounding related to alcohol consumption behaviours cannot be completely excluded (Hernan and Robins, 2016). Third, the UK Biobank is predominantly composed of White participants, and previous polygenic-score studies have emphasized that predictive performance and generalizability may be limited across ancestries (Martin et al., 2019). Although we used the enhanced ischemic stroke PRS released by the UK Biobank, the present Cox models did not additionally adjust for genetic principal components, genotyping array, or relatedness. Because PRS associations may be influenced by population stratification and ancestry structure, residual confounding related to genetic ancestry cannot be completely excluded. Therefore, the PRS-related findings should be interpreted cautiously and require validation in future studies with comprehensive genetic quality-control adjustment and more diverse ancestry groups. Furthermore, the PRS-related analyses were restricted to participants with available genetic data and required model information; therefore, they included substantially fewer ischemic stroke events than the main physical activity analysis. This reduced statistical power, particularly for the joint analysis across the nine PRS–physical activity categories, and may have contributed to wider confidence intervals and non-significant estimates in some categories. Accordingly, the PRS-only and joint PRS–physical activity findings should be regarded as exploratory and require confirmation in larger studies with more complete genetic and accelerometer data. In addition, UK Biobank participants are generally healthier and may have more favorable socioeconomic and lifestyle characteristics than the general population. Furthermore, the present analysis was restricted to participants with valid accelerometer-derived physical activity data and complete covariate information. Participants excluded because of missing accelerometer data or incomplete covariate information may have differed from the included analytic sample in baseline demographic, socioeconomic, lifestyle, or clinical characteristics. Therefore, selection bias and healthy volunteer bias cannot be fully excluded, and the absence of such bias could not be confirmed in the present study. Therefore, caution is warranted when extrapolating the present findings to other ethnic groups, or the broader UK Biobank population without valid accelerometer data. Fourth, the proportional hazards assumption was not formally tested in the current analytic workflow. Although the joint-category analyses provided information on the combined associations of physical activity patterns and polygenic susceptibility with ischemic stroke risk, formal interaction analyses on either the multiplicative or additive scale were not performed, and no p-interaction value was estimated. Therefore, these findings should be interpreted as descriptive joint associations rather than evidence that physical activity statistically modifies or attenuates genetic risk. Fifth, existing evidence suggests that the incremental predictive value of current stroke polygenic scores over traditional risk factors remains modest, so the clinical translation of combined behavioral-genetic risk stratification still requires further validation (Abraham et al., 2019).

Overall, the current evidence supports the importance of both sufficient weekly physical activity and genetic susceptibility in ischemic stroke risk assessment. Our findings extend this literature by suggesting that objectively measured physical activity pattern and polygenic risk may be considered jointly when evaluating incident ischemic stroke risk.

## Conclusion

In summary, in this prospective cohort study using accelerometer-derived physical activity data, both physical activity patterns and genetic susceptibility were associated with incident ischemic stroke. Participants who achieved the recommended weekly level of moderate-to-vigorous physical activity had a lower risk of ischemic stroke compared with inactive individuals, and no statistically significant difference was observed between the active weekend warrior and active regular groups. Greater polygenic risk was associated with a higher risk of ischemic stroke. Joint-category analyses further suggested that participants with physical inactivity and higher genetic susceptibility had the highest risk estimates. Active regular and active weekend warrior patterns did not show statistically significant protective associations within the high genetic risk group. Therefore, these joint findings should be interpreted as descriptive associations rather than evidence of formal interaction or attenuation of genetic risk. Overall, objectively measured physical activity patterns and genetic susceptibility may be considered together when characterizing ischemic stroke risk, but further studies are needed to confirm these associations and clarify the underlying mechanisms.

## Data Availability

The datasets presented in this study can be found in online repositories. The names of the repository/repositories and accession number(s) can be found in the article/Supplementary material.
