# Supplementary material for: Physical activity patterns, genetic susceptibility, and incident ischemic stroke: a population-based cohort study
Source: Front Aging Neurosci. 2026 May 29;18:1840853. doi: 10.3389/fnagi.2026.1840853 (PMC13260129; doi:10.3389/fnagi.2026.1840853)
Supplement: Supplementary file 1 [file Data_Sheet_1.DOCX]

Supplementary Table S1

Joint Categories of Genetic Risk and Physical Activity Pattern and Risk of Stroke (MVPA threshold = 101 min/week)

| Genetic Risk + Physical Activity | No of participants | No of events | HR (95%CI) |
| --- | --- | --- | --- |
| Low Genetic Risk + Active Regular | 1919 | 16 | ref |
| Low Genetic Risk + Inactive | 2087 | 28 | 1.59 (0.86-2.96, p=0.14) |
| Low Genetic Risk + Active WW | 4096 | 36 | 1.01 (0.56-1.82, p=0.98) |
| Intermediate Genetic Risk + Active Regular | 1696 | 15 | 1.06 (0.53-2.15, p=0.86) |
| Intermediate Genetic Risk + Inactive | 2084 | 30 | 1.75 (0.95-3.22, p=0.08) |
| Intermediate Genetic Risk + Active WW | 3746 | 43 | 1.32 (0.74-2.35, p=0.34) |
| High Genetic Risk + Active Regular | 1468 | 24 | 2.02 (1.07-3.80, p<0.05) |
| High Genetic Risk + Inactive | 2093 | 43 | 2.56 (1.43-4.58, p<0.01) |
| High Genetic Risk + Active WW | 3430 | 50 | 1.72 (0.98-3.02, p=0.06) |

Genetic risk was categorized into low, intermediate, and high groups according to polygenic risk score tertiles. Physical activity pattern was classified as active regular, inactive, and weekend warrior (WW). The joint categories were defined by combining genetic risk groups and physical activity patterns. The reference group was participants with low genetic risk and regular physical activity. The threshold for MVPA was 101 min/week.

Supplementary Table S2

Joint Categories of Genetic Risk and Physical Activity Pattern and Risk of Stroke (MVPA threshold = 230 min/week)

| Genetic Risk + Physical Activity | No of participants | No of events | HR (95%CI) |
| --- | --- | --- | --- |
| Low Genetic Risk + Active Regular | 1667 | 12 | ref |
| Low Genetic Risk + Inactive | 4131 | 46 | 1.55 (0.82-2.94, p=0.18) |
| Low Genetic Risk + Active WW | 2304 | 22 | 1.21 (0.60-2.45, p=0.59) |
| Intermediate Genetic Risk + Active Regular | 1452 | 11 | 1.04 (0.46-2.36, p=0.92) |
| Intermediate Genetic Risk + Inactive | 4091 | 56 | 1.92 (1.02-3.60, p<0.05) |
| Intermediate Genetic Risk + Active WW | 1983 | 21 | 1.37 (0.67-2.79, p=0.38) |
| High Genetic Risk + Active Regular | 1232 | 18 | 2.05 (0.99-4.26, p=0.05) |
| High Genetic Risk + Inactive | 3892 | 67 | 2.48 (1.34-4.62, p<0.01) |
| High Genetic Risk + Active WW | 1867 | 32 | 2.25 (1.16-4.38, p<0.05) |

Genetic risk was categorized into low, intermediate, and high groups according to polygenic risk score tertiles. Physical activity pattern was classified as active regular, inactive, and weekend warrior (WW). The joint categories were defined by combining genetic risk groups and physical activity patterns. The reference group was participants with low genetic risk and regular physical activity. The threshold for MVPA was 230 min/week.

Supplementary Table S3

Joint Categories of Genetic Risk and Physical Activity Pattern and Risk of Stroke (MVPA threshold = 403 min/week)

| Genetic Risk + Physical Activity | No of participants | No of events | HR (95%CI) |
| --- | --- | --- | --- |
| Low Genetic Risk + Active Regular | 999 | 3 | ref |
| Low Genetic Risk + Inactive | 6247 | 68 | 3.63 (1.14-11.56, p<0.05) |
| Low Genetic Risk + Active WW | 936 | 9 | 2.83 (0.77-10.46, p=0.12) |
| Intermediate Genetic Risk + Active Regular | 6 | 6 | 2.18 (0.55-8.73, p=0.27) |
| Intermediate Genetic Risk + Inactive | 878 | 72 | 4.08 (1.28-12.97, p<0.05) |
| Intermediate Genetic Risk + Active WW | 757 | 10 | 3.92 (1.08-14.27, p<0.05) |
| High Genetic Risk + Active Regular | 725 | 10 | 4.59 (1.26-16.67, p<0.05) |
| High Genetic Risk + Inactive | 5634 | 98 | 6.07 (1.92-19.21, p<0.01) |
| High Genetic Risk + Active WW | 749 | 9 | 3.57 (0.97-13.20, p=0.06) |

Genetic risk was categorized into low, intermediate, and high groups according to polygenic risk score tertiles. Physical activity pattern was classified as active regular, inactive, and weekend warrior (WW). The joint categories were defined by combining genetic risk groups and physical activity patterns. The reference group was participants with low genetic risk and regular physical activity. The threshold for MVPA was 403 min/week.
